# Anticancer efficacy of 3-(4-isopropyl) benzylidene-8-ethoxy, 6-methyl, chroman-4-one (SBL-060), a novel, dual, estrogen receptor-Akt kinase inhibitor in acute myeloid leukemia cells

**DOI:** 10.32604/or.2022.03539

**Published:** 2022-08-01

**Authors:** MESFER AL SHAHRANI, PRASANNA RAJAGOPALAN, MOHAMMAD ABOHASSAN, MOHAMMAD ALSHAHRANI, YASSER ALRAEY, REEM M. GAHTANI, SURESH RADHAKRISHNAN, KHLOOD DAGREERY

**Affiliations:** 1 Department of Clinical Laboratory Sciences, College of Applied Medical Sciences, King Khalid University, Abha, Saudi Arabia; 2 Central Research Laboratory, College of Applied Medical Sciences, King Khalid University, Abha, Saudi Arabia; 3 PG and Research Department of Chemistry, Presidency College, Chennai, Tamil Nadu, India; 4 Regional Laboratory and Central Blood Bank, Jazan, Saudi Arabia

**Keywords:** Akt kinase, AML, THP-1, HL-60, Estrogen receptor, Benzylidene compounds

## Abstract

Estrogen receptor (ER) α is expressed in a subset of patient-derived acute myeloid leukemia (AML) cells, whereas Akt is predominantly expressed in most types of AML. Targeting AML with dual inhibitors is a novel approach to combat the disease. Herein, we examined a novel small molecule, 3-(4-isopropyl) benzylidene-8-ethoxy,6-methyl, chroman-4-one (SBL-060), capable of targeting AML cells by inhibiting ERα and Akt kinase. The chemical properties of SBL-060 were identified by proton nuclear magnetic resonance (^1^H-NMR), ^13^C-NMR, and mass spectroscopy. *In silico* docking was performed using an automated protocol with AutoDock-VINA. THP-1 and HL-60 cell lines were differentiated using phorbol 12-myristate 13-acetate. ERα inhibition was assessed using ELISA. The MTT assay assessed cell viability. Flow cytometry was performed for cell cycle, apoptosis, and p-Akt analyses. Chemical analysis identified the compound as 3-(4-isopropyl) benzylidene-8-ethoxy,6-methyl, chroman-4-one, which showed high binding efficacy toward ER, with a ΔG_binding_ score of −7.4 kcal/mol. SBL-060 inhibited ERα, exhibiting IC_50_ values of 448 and 374.3 nM in THP-1 and HL-60 cells, respectively. Regarding inhibited cell proliferation, GI_50_ values of SBL-060 were 244.1 and 189.9 nM for THP-1 and HL-60 cells, respectively. In addition, a dose-dependent increase in sub G_0_/G_1_ phase cell cycle arrest and total apoptosis was observed after treatment with SBL-060 in both cell types. SBL-060 also dose-dependently increased the p-Akt-positive populations in both THP-1 and HL-60 cells. Our results indicate that SBL-060 has excellent efficacy against differentiated AML cell types by inhibiting ER and Akt kinase, warranting further preclinical evaluations.

## Introduction

Acute myeloid leukemia (AML) is an aggressive form of hematologic malignancy induced by myeloid cell (immature) accretion in the blood/bone marrow [1]. AML can be attributed to unusual genetic mutations that accumulate with chromosomal translocations and/or epigenetic modifications [2]. These factors render the disease unique from patient to patient, consequently posing a challenge to treat. Current therapies to treat AML include combined cytarabine and anthracycline, affording an average success rate of 35%–45% in patients aged <60 years and 10%–15% in patients aged >60 years [3]. Therefore, novel agents that are more efficacious against AML are urgently needed. The 3-benzylidene chroman-4-one class of compounds shares a close homology with naturally occurring bioactive compounds such as flavanones, flavones, chromones, and coumarins. Given the common occurrence of basic side chains in therapeutically active compounds, it is deemed worthwhile to incorporate basic groups into chromanones and evaluate their biological activities. Hence, we synthesized such analogs to screen their bioactivities.

AML is often characterized by myeloblast-induced clonal expansion [4]. As this form of the disease has a stem cell-derived hematopoietic origin, uncontrolled accumulation of these cells can be fatal [5]. Estrogen receptor (ER) α and Erβ are encoded by *ESR1* and *ESR2* genes, respectively, and mediate estrogen signaling [6]. In addition, the pattern of distribution of these ERs has recently gained momentum as novel targets in various diseases [1]. In terms of malignancies, it has been reported that Erα expression increases with for potential involvement in disease etiology and progression [7]. Therefore, targeting ERs in AML cells could afford a reasonable target to combat disease progression. Tumor progression and hence is regarded as a valid target to control rapidly proliferating cancer cells [8]. Although AML is not directly linked to sex hormones, the relationship between estrogen, ERs, and AML has been documented [2]. Accumulated evidence suggests that males are twice as likely to be diagnosed with AML than females, suggesting the involvement of estrogen in the disease [3]. The expression of ERs in differentiated AML cells and aberrant hematopoiesis has been assessed. In contrast, inhibitors of Akt kinase can reportedly control the proliferation of AML cells [4]. However, targeting AML cells using a dual ER and Akt inhibitor has not been evaluated. Our previous work shows structure activity relationship of 3-Benzylidene Chroman-4-one analogues to be possess antimicrobial and anticancer efficacies targeting ER and Akt [5]. Accordingly, the present study focused on the efficacy of one such novel small molecule, 3-(4-isopropyl) benzylidene-8-ethoxy,6-methyl, chroman-4-one (SBL-060), against AML cells by utilizing computational and *in vitro* approaches.

## Materials and Methods

### Materials

Chemicals were purchased from Sigma-Aldrich Aldrich USA. THP-1 (acute monocytic leukaemia) and HL-60 (acute promyelocytic leukaemia) cell lines were obtained from the American Type Culture Collection (Rockville, MD, USA). Annexin V and cell cycle assay reagents were purchased from Merck Millipore, CA, USA. Anti-p-Akt-PE antibody was obtained from eBioscience (Thermo Scientific Corp., MA, USA). The ER-alpha ELISA kit was purchased from BioVision Corp., CA, USA.

### Methods

#### Chemical synthesis

**Figure 1:**
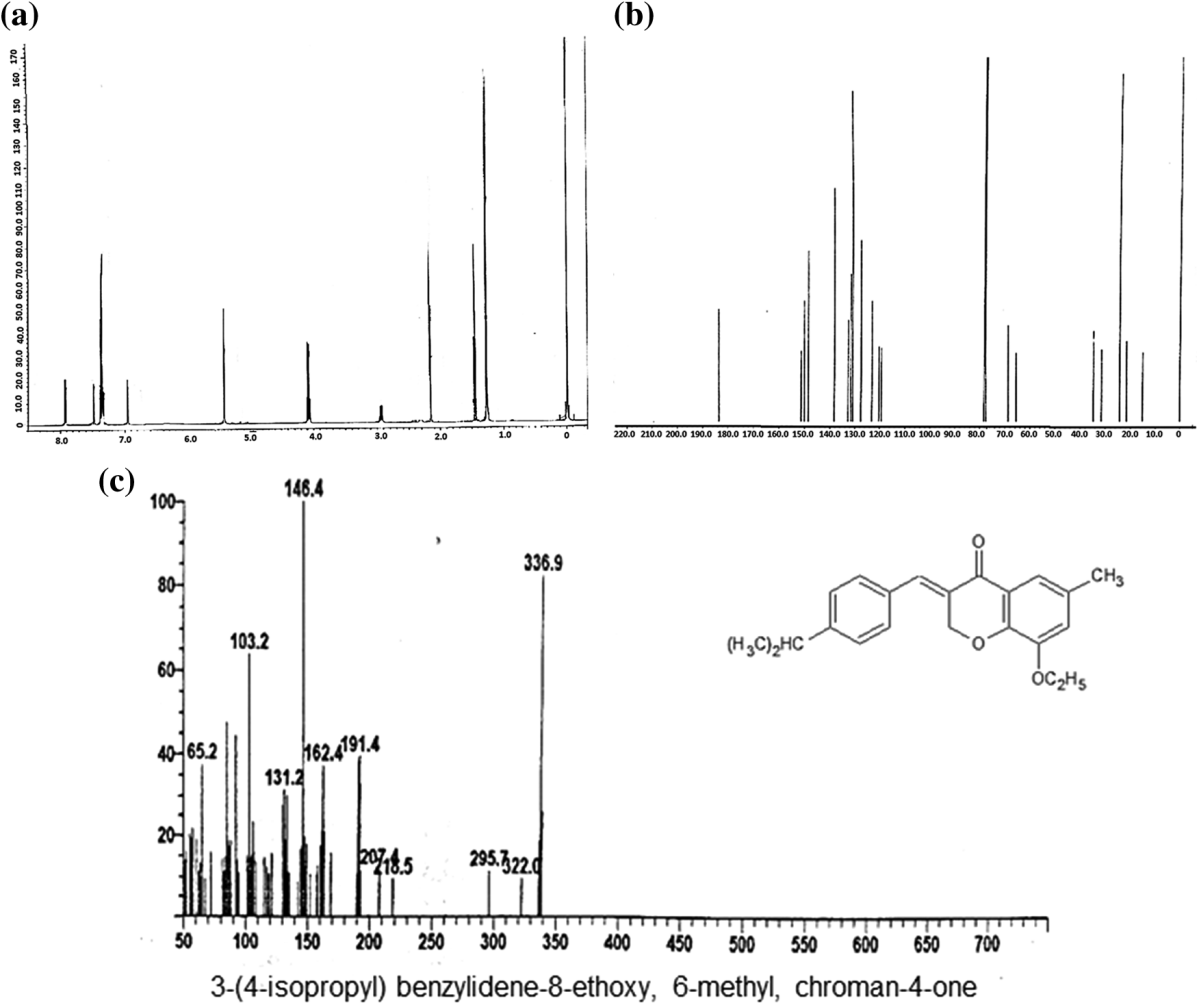
Chemical Properties for synthesized compound, i.e., 3-(4-isopropyl) benzylidene-8-ethoxy,6-methyl, chroman-4-one (SBL-060). (a) Histogram of the proton nuclear magnetic resonance spectroscopy (NMR) for SBL-060. (b) Carbon NMR of SBL-060. (c) Mass spectroscopy of SBL-060 elucidating the chemical properties of SBL-060. (d) δ values based on NMR results and elemental analysis of the compound.

3-(4-Isopropyl) benzylidene-8-ethoxy,6-methyl, chroman-4-one (Fig. 1) was synthesized in-house. Briefly, the synthesis of 3-benzylidene-8-ethoxy,6-methyl, chroman-4-one was initiated using benzaldehyde and methyl acrylate. The Baylis–Hillman reaction, with DABCO as a catalyst, was performed in the solid phase using silica gel in the absence of any solvent, generating methyl-a-methylene-b-hydroxy-b-phenyl propanoate. Hydroxypropanoate was treated with hydrobromic acid with a catalytic amount of concentrated sulfuric acid at room temperature to yield bromomethyl propenoate. Then, bromomethyl propenoate was treated with eugenol in the presence of potassium carbonate in acetone to yield methyl 3-aryl-2-(2-methoxy-4-prop-2-enyl) phenoxy ethyl prop-2-enoate. The ester was hydrolyzed using potassium hydroxide in aqueous 1,4-dioxane at room temperature. The acid was obtained by acidification, generating 3-aryl-2 [2-methoxy-4(prop-2-enyl)] phenoxy methyl prop-2-enoic acid. Propenoic acid was subjected to intramolecular Friedel–Crafts acylation by reacting with trifluoroacetic anhydride (TFAA) in methylene dichloride to generate the desired product.

#### Structure retrieval and processing

The X-ray crystal structures of ERs (PDBid: 1UOM) and AKT (PDB # 6HHG) were retrieved from the PDB databank (www.rcsb.org). Before docking, all receptor structures were processed by removing external water and adding hydrogens using BIOVIA-Discovery Studio Visualizer. In addition, the SBL-060 structure in SDF format was converted to SYBYL-TRIPOS (mol2) format using BIOVIA-Discovery Studio Visualizer. Likewise, all target unknown ligand structures were prepared using the BIOVIA-Discovery Studio Visualizer prior to docking analysis.

#### Computational docking analysis

Docking was performed using the SiBDOCk (automated docking submission module) developed by SiBIOLEAD (https://sibiolead.com/). Briefly, a docking box was generated based on the information obtained from respective protein structures, complexed with known inhibitors by selecting two amino acid residues on either side of the active site. The AutoDock-VINA program was used with the standard docking mode. The top ligands were ranked based on the docking scores. Protein-ligand interactions were inferred using the “Receptor-ligand Interactions” module in BIOVIA-Discovery Studio Visualizer.

#### Cell culture, ER inhibition and cell proliferation assays

RPMI-1640 medium was used to culture THP-1 and HL-60 cells. Cell culture was performed using standard protocols. The growth medium contained 10% fetal bovine serum (FBS), 100 U/mL of penicillin, and 100 U/mL streptomycin. Differentiation with phorbol 12-myristate 13-acetate (PMA) can reportedly increase ER expression in AML cells [6]. Prior to the *in vitro* assays, both THP-1 and HL-60 cells were differentiated by adding 50 ng/mL PMA and further incubated for 48 h [7]. Subsequently, the cells were centrifuged and re-plated to perform the different assays. For ER-ELISA, differentiated THP-1 and HL-60 cells were plated in 24-well plates, treated with different concentrations of SBL-060, and incubated for 24 h. Cellular supernatants were collected and centrifuged for 20 min at 1000 g at 4°C to remove debris, and a clear solution was used as the sample. ELISA was performed according to the manufacturer’s instructions. Cell proliferation was assessed using the MTT assay, as described previously [8]. Briefly, THP-1 and HL-60 cells at a concentration of 5 × 10^3^ cells/well were grown in 96-well tissue culture plates in regular growth medium. The cells were then treated with different concentrations of the test compound for 24 h. After removing the medium, 100 µL of MTT (1 mg/mL) was added as a medium replacement and incubated for 4 h. Formazan products were dissolved in 200 µL dimethyl sulfoxide (DMSO), and absorbance was measured at 560 nm. Percent inhibition was calculated using GraphPad Prism 6.0 (GraphPad Software, Inc., La Jolla, USA).

#### Flow cytometry analysis for cell cycle and apoptosis

The cell cycle assay was performed using the cell cycle assay kit according to the manufacturer’s instructions. Differentiated THP-1 and HL-60 cells, at a density of 5 × 10^5^ cells per well in a 6-well plate, were treated with 100, 200, and 400 nM SBL-060 and further incubated for 72 h. After washing cells with phosphate-buffered buffer (PBS) twice, 50 µL of cell cycle assay reagent was added, followed incubation for 15 min in the dark. After washing with PBS to remove excess staining reagent, the cells were resuspended in Hank’s balanced salt solution (HBSS) buffer. Then, 10,000 events were acquired on a Guava easyCyte™ flow cytometer, and data were analyzed with ExpressPro Software from Millipore (Burlington, CA, USA). The percentage of cell populations in the sub G_0_.G_1_ phase of the cell cycle was determined. The apoptosis assay was performed using an Annexin V detection kit in accordance with the manufacturer’s instructions. After differentiation, both AML cell lines were treated as indicated for the cell cycle assay and incubated for 48 h. Then, 0.25 µg/mL Annexin V reagent was added to cells for 15 min in the dark. After two washes using sterile PBS, cells were resuspended in kit buffer containing 0.5 µg/mL propidium iodide. Next, 10,000 events were acquired on a Guava easyCyte™ flow cytometer. Data analysis was performed using InCyte software to differentiate between healthy and apoptotic cells (early and late apoptosis) and presented using GraphPad Prism version 6.0.

#### Akt inhibition assay by flow cytometry

Differentiated THP-1 and HL-60 cells were plated in 6-well plates with complete media and treated with 100, 200 or 400 nM SBL-060 for 4 h. Then, cells were twice-washed with PBS, followed by the addition of 0.25 μg/mL PE-conjugated, anti-p Akt antibody and incubation for 20 min in the dark. After a couple of washes in PBS, the cells were resuspended in HBSS buffer, and 10,000 events were acquired using a Guava easyCyte™ flow cytometer. Data were analyzed using InCyte software from Millipore (Burlington, CA, USA). The percentage of positive p-Akt cells was estimated.

#### Statistical analysis

Statistical analyses were performed using GraphPad Prism 6.0. Results are expressed as mean ± standard error (SE). Data were analyzed using ANOVA followed by multiple comparisons. Statistical significance was set at *p* ≤ 0.05.

## Results

### Chemical elucidations for the synthesized compound

The IR spectrum (Fig. 1a and Table 1) shows absorption at 16653 and 1600 cm^–1^, representing an aromatic α,β-unsaturated ketone. The PMR spectrum shows a multiplet around 7.20–7.22 δ for seven protons (Fig. 1b and Table 1). The m/e value of the compound corresponds to the molecular weight of 336.2 (Fig. 1c and Table 1) and elemental analysis agrees with the molecular formula of a compound. Calcd: C, 78.63%; H, 7.20%; Found: C, 78.72%; H, 7.22%. (Table 1). Based on the above data, the compound was identified as 3-(4-isopropyl) benzylidene-8-ethoxy,6-methyl, chroman-4-one (Fig. 1c), internal reference code for the compound SBL-060. The compound had 91% purity as determined by 1H NMR.

**Table 1:** Elemental analysis and chemical characteristics depicting physical and chemical properties of SBL-060 based on NMR and mass spectroscopy results

^1^H NMR			
Substrate (R)	Melting point (°C)	^1^H NMR (δ)	IR (cm^−1^)
CH(CH_3_)_2_	141	7.84 (s, 1H), 7.25–7.4 (q, 4H, J = 7.8 Hz), 7.45 (d, 1H, J = 7.6 Hz), 6.9 (s, 1H), 5.4 (d, 2H, J = 1.6 Hz), 4.08 (q, 2H, J = 7.2 Hz), 2.93 (m, 1H), 2.29 (s, 3H), 1.45 (d, 3H, J = 7.2 Hz),	1665 1600
		1.27 (d, 6H, J = 7.0 Hz)	
**Aromatic region (δ)**	**H**_**a**_ **(δ)**	**H**_**b**_ **(δ)**	**Ar–CH**_**3**_ **(δ)**
7.45 (d, 1H, J = 7.6 Hz), 7.25–7.4 (q, 4H, J = 7.8 Hz), 6.9 (s, 1H),	7.84 (d, 2H)	5.4 (d, 2H, J = 1.6 Hz)	2.29 (s, 3H)
^**13**^ **C NMR**			
**δ values (ppm)**			
21.19, 23.86, 24.08, 343.14, 56.34, 77.20, 77.45, 81.87, 118.62, 122.35, 126.95, 127.53, 130.35, 131.99, 137.67, 148.56, 149.17, 154.72, 176.74, 182.52			

**Mass spectrum**						
**Yeild (%)**	**Melting point (°C)**	**IR (cm** ^**−1**^ **)**	**MS** **(70 ev)** **M/Z (M**^**+**^**)**	**Molecular formula**	**Elemental analysis (%)**	
					**Cacld/Found**	
91	141	1665, 1660	336.2	C_22_H_24_O_3_	**C**	**H**
					**78.63**	**7.20**
					**78.72**	**7.22**

### SBL-060 was potently bound to the ER crystal structure

**Figure 2:**
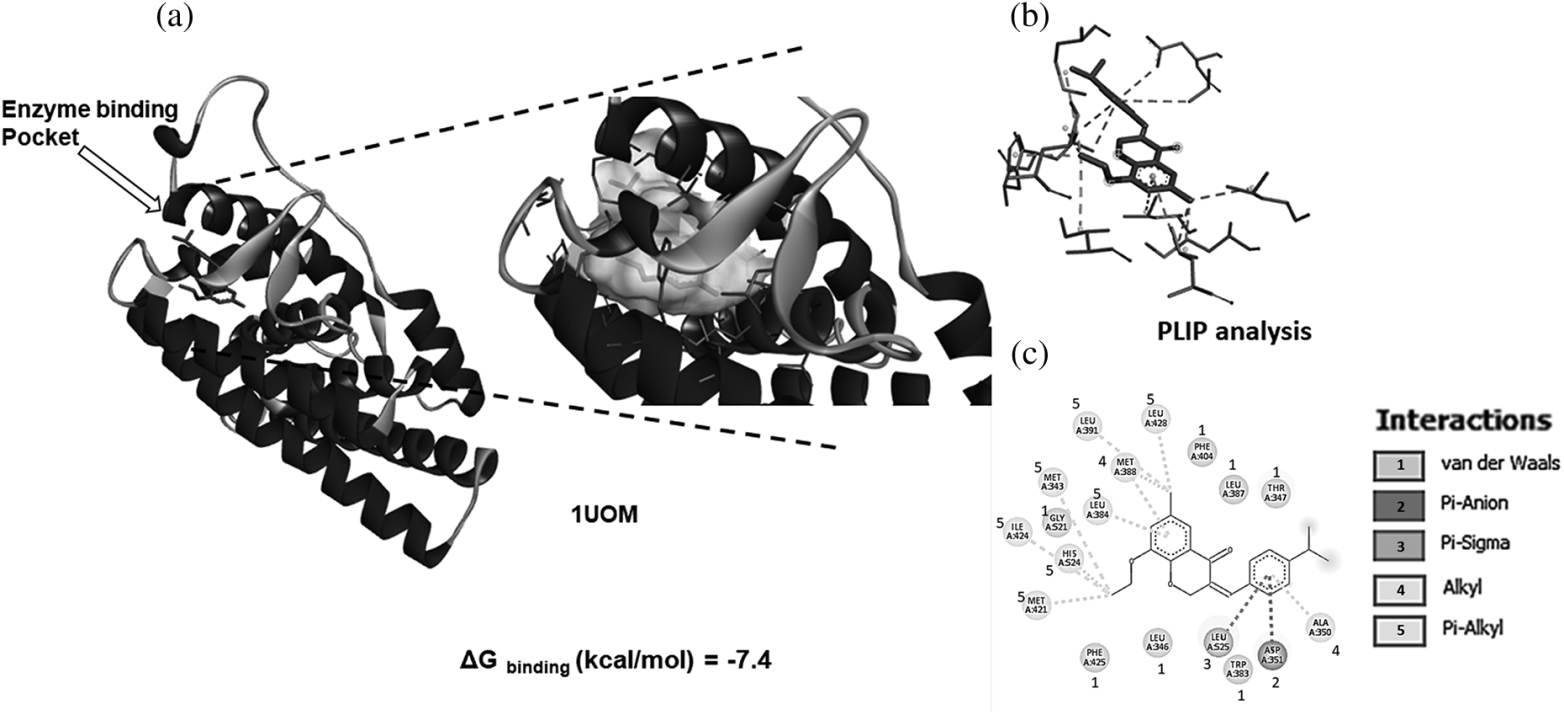
Computational docking efficacy of SBL-060 to the crystal structure of ER (1UOM). (a) Full-length ER protein docks with SBL-060. The arrow indicates the active binding pocket of the enzyme. A magnified image showing the ligand-binding position and interactions within the active site. (b) Image showing SBL-060 associated ligand-protein interactions. (c) A 2-dimensional analysis of the protein-ligand amino acid interactions involved in SBL-060 and ER binding. ER, estrogen receptor.

Using a computational approach, we evaluated the docking efficacy of SBL-060 with the protein ER. As shown in Fig. 2a, the compound was bound to the energy-binding pocket of the protein with high affinity. On analyzing the binding energy, SBL-060 exhibited docking energy of −7.4 kcal/mol (Fig. 2a). Protein-ligand interaction profiler (PLIP) analysis (Fig. 2b) of critical interacting residues revealed LEU391, LEU428, MET388, LEU384, MET343, GLY52, ILE424, HIS524, MET421, LEU525, ASP351, and ALA350 (Fig. 2c).

### Inhibition of ER and cell proliferation by SBL-060 in differentiated AML cells

**Figure 3:**
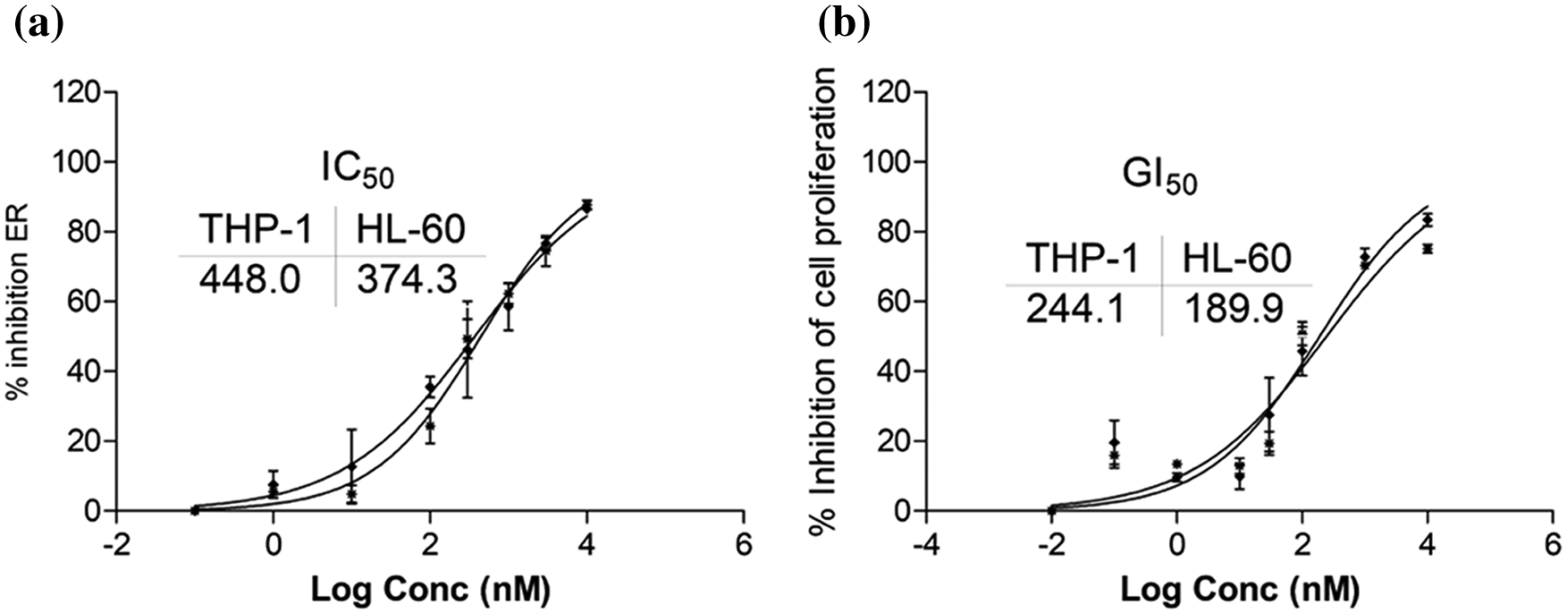
*In vitro* efficacy evaluation of SBL-060 in differentiated AML cells (a) shows the IC_50_ of SBL-060 for ER inhibition in the differentiated THP-1 and HL-60 cells. (b) The antiproliferative efficacy of SBL-060 with respective GI_50s_ in PMA-differentiated THP-1 and HL-60 cells. ER, estrogen receptor; PMA, phorbol 12-myristate 13-acetate. Data were mean ±SE (n = 3).

To mimic ERα expression in AML, we used PMA to induce ER and subsequently assessed the activity of SBL-060. We observed that SBL-060 dose-dependently inhibited ERα in differentiated AML cells, with IC_50_ (half maximal inhibitory concentration) values of 488.0 and 374.3 nM in THP-1 and HL-60 cells, respectively (Fig. 3a). Additionally, SBL-060 inhibited the proliferation of THP-1 and HL-60 cells, with GI_50_ (half maximal growth inhibitory concentration) values of 244.1 and 189.9 nM, respectively (Fig. 3b). Cytarabine was used as a standard and the activity is provided as Suppl. Fig. 1.

### SBL-060 increased the sub G_0_/G_1_ cell populations in AML cells

**Figure 4:**
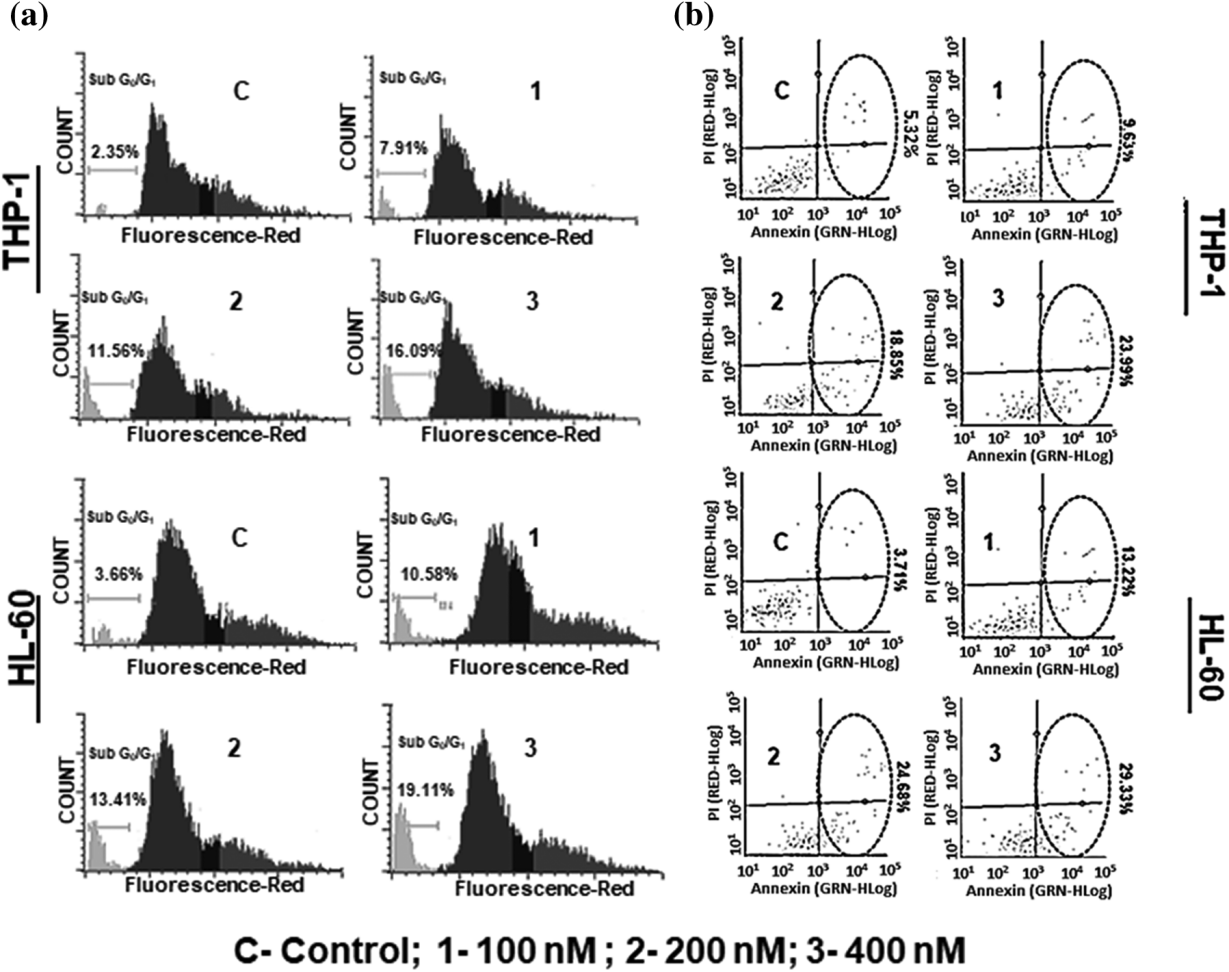
Functional cellular assays demonstrating the effect of SBL-060 in THP-1 and HL-60 cells. (a) Representative histograms of cell cycle analysis in both the differentiated AML cells with different SBL-060 dose treatments. A dose-based increase in the sub G_0_/G_1_ phase of the cell cycle can be observed in both THP-1 and HL-60 cells at the end of 72 h. Data values are mean ± standard deviation (SD) percentage of sub G_0_/G_1_ phase cells from different experiments. (b) Early and late phase apoptotic cell populations in THP-1 and HL-60 cells treated with different SBL-060 doses for 48 h. Representative plots are presented. The lower right quadrant indicates early apoptotic cells, and the upper right quadrant represents late apoptotic cells. Numerical values are the total apoptotic cells, presented as mean ± SD from different experiments.

To assess the efficacy of SBL-060 in cellular functional assays, we examined the effect of SBL-060 on cell cycle changes and apoptosis induction in PMA-differentiated THP-1 and HL-60 cells. We selected three doses (low and high) based on the proliferation inhibition results of SBL-060 in these cells. Following treatment with SBL-060, we observed a dose-dependent increase in the sub G_0_/G_1_ phase in both AML cell lines (Fig. 4a). Compared with untreated control cells, THP-1 cells showed an increase in the sub G_0_/G_1_ population from 2.35% to 7.91% following treatment with 100 nM SBL-060 (Fig. 4a). Following treatment with 200 and 400 nM SBL-060, the percentage of THP-1 cells in the sub G_0_/G_1_ phase increased to 11.56% and 16.09%, respectively (Fig. 4a). Similarly, untreated HL-60 cells exhibited a sub G_0_/G_1_ population of 3.66%, and treatment with 100, 200, and 400 nM SBL-160 increased the percentage to 10.58%, 13.41%, and 19.11%, respectively (Fig. 4a).

### Induction of total apoptosis in THP-1 and HL-60 cells by SBL-060

On analyzing total apoptosis in AML cells, both early and late phase apoptosis were increased with different SBL-060 doses in both cell lines (Fig. 4b). In the untreated control THP-1 and HL-60 cells, the percentage of total apoptotic cells was 5.23% and 3.71%, respectively (Fig. 4b). Treatment with 100, 200, and 400 nM SBL-060 increased the total apoptotic cell percentage to 9.63%, 18.85%, and 23.99% in THP-1 cells and 13.22%, 24.68%, and 29.33% in HL-60 cells (Fig. 4b).

### SBL-060 bound and inhibited the Akt enzyme

**Figure 5:**
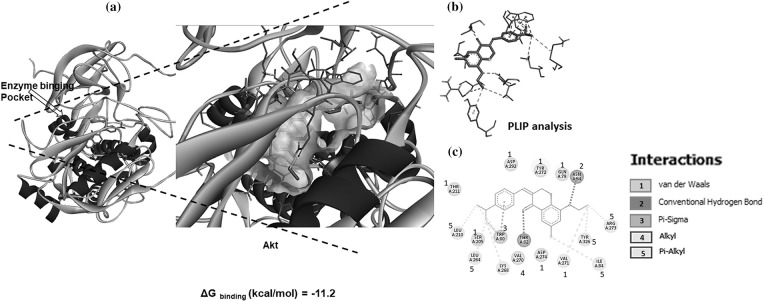
*In silico* docking of SBL-060 to the crystal structure of the Akt enzyme. (a) Representative image of the full-length Akt enzyme bound with SBL-060 in the active binding pocket of the enzyme, as indicated by an arrow. The magnified image shows the ligand-binding position and interactions within the active site. (b) Image showing SBL-060 associated ligand-protein interactions. (c) A 2-dimensional analysis of the protein-ligand amino acid interactions involved in SBL-060 binding with Akt.

**Figure 6:**
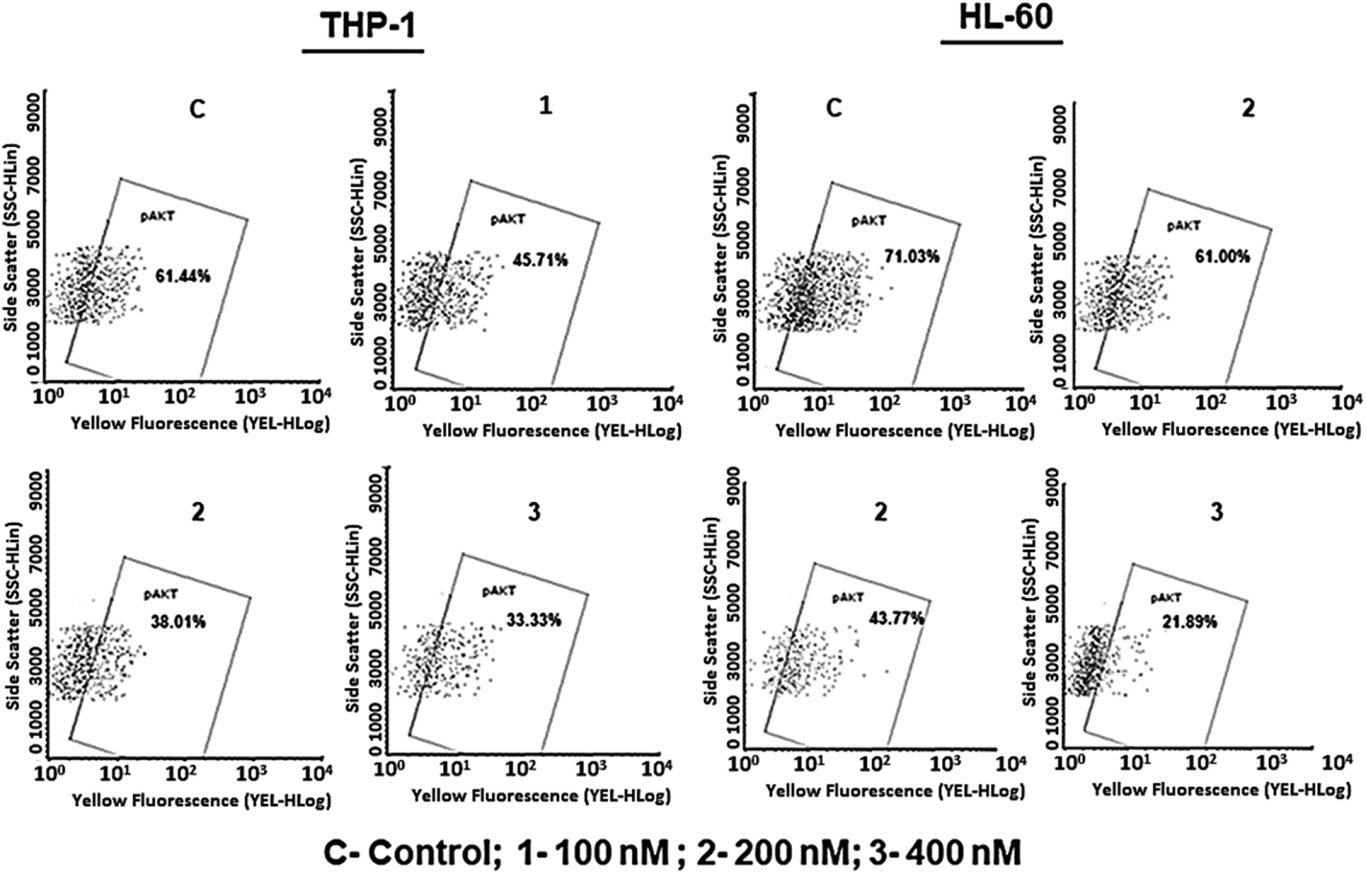
Flow cytometric enumeration of the percentage Akt-positive cells in THP-1 and HL-60 cells. The numerical values indicated are mean ± standard deviation (SD) from different experiments. A dose-dependent reduction in the Akt-positive cells can be observed in both AML cell lines following SBL-060 treatment.

Next, we determined whether SBL-060 could inhibit the Akt enzyme, known to be predominantly expressed during AML malignancy. Prior to the *in vitro* testing, we performed computational *in silico* docking to assess the binding affinity of SBL-060 toward Akt. Based on our findings, SBL-060 presented an excellent binding affinity to the enzyme, exhibiting a docking score of −11.2 kcal/mol (Fig. 5a). Based on the PLIP analysis, the chief interacting amino acids were ASN54, SER205, LEU210, TRP80, LYS268, THR82, VAL271, TYR326, ILE84, and ARG 273 (Figs. 5b and 5c). To determine if this docking efficacy was translated *in vitro*, Akt inhibition was assessed by flow cytometry. The Akt-positive population in untreated THP-1 cells was 61.44% (Fig. 6). Following treatment with 100, 200, and 400 nM SBL-060, the percentage of positive Akt cells decreased to 45.71%, 38.01%, and 33.33%, respectively (Fig. 6). In HL-60 cells, the percentage of Akt-positive cells was 71.03% in the untreated control; this percentage reduced to 61.00%, 43.77%, and 21.89% following treatment with 100, 200, and 400 nM SBL-060, respectively (Fig. 6).

## Discussion

ER and Akt are widely activated in breast cancer types and remains as an attracting target to control breast cancer proliferations [9,10]. We have earlier shown novel 3-Benzylidene Chroman-4-one analogues to effectively control breast cancer proliferation targeting ER and Akt enzyme [5]. Accumulated evidence suggests a strong correlation between ERα expression in a subset of patient-derived AML cells [11,12]. Methylation of ERα is frequently observed in normal AML karyotypes, which leads to repressed ERα gene transcription [13]. In addition, hypermethylation of ERα has been shown to improve AML survival rates [14], which indirectly indicates that ERα expression is positively correlated with disease progression. Remarkably, of the 54 genes known to drive AML progression, 35% are reportedly associated and regulated by ERα and E2 [15]. Attempts to regulate AML using ERα antagonists, such as tamoxifen, were found to be successful [16]. The binding position and computational docking score of SBL-060 were satisfactory, necessitating further *in vitro* evaluations of the compound. Consistent with computational observations, SBL-060 effectively inhibited ERα in a dose-dependent manner. This activity was also supported by GI_50_ values of SBL-060 in the nano-molar range, as determined by antiproliferative assays against THP-1 and HL-60 cells.

Cell cycle and apoptosis assays were performed to further evaluate the effects of SBL-060 on the functionality of AML cells. The cell cycle is considered an important checkpoint for rapidly proliferating cells to determine life and death [17]. An increase in the sub G_0_/G_1_ phase of AML cells has been associated with successful treatment of AML *in vitro* [18]. Furthermore, benzylidene chroman-4-ones were shown to induce sub G_0_/G_1_ phase accumulation during cancer cell treatments [19]. Therefore, our data regarding the dose-dependent increase in the sub G_0_/G_1_ phase of AML cells following SBL-060 treatment agreed with the aforementioned reports. Moreover, ERα antagonists have been shown to control AML progression via induction of apoptosis [20]. Consistent with these studies, SBL-060 successfully induced dose-dependent apoptosis in both AML cell types examined.

Protein kinase B (Akt) is a serine-threonine kinase of the PI3K family [21]. Akt is a pivotal regulator of several key cell survival functions, including growth, cell division, angiogenesis, and suppression of apoptosis [22]. Akt dysregulation is frequently observed in numerous human cancer types, and several drug discovery efforts have focused on the development of Akt inhibitors [23]. In most patients with AML, constitutional Akt phosphorylation reportedly occurs via the PI3K-Akt-mTOR pathway, an important signal for AML cell survival [24,25]. Furthermore, studies have revealed a close homology between benzylidene compounds and target Akt for modulating cancer proliferation [26]. Therefore, computational screening of SBL-060 against the Akt enzyme should be further explored. Our docking scores strongly suggest the involvement of Akt inhibition by SBL-060. This computational efficacy was undoubtedly translated in our *in vitro* findings, exhibiting the dose-dependent inhibition of Akt phosphorylation by SBL-060 in both AML cell types. Previous studies have shown that cell cycle alterations and apoptosis induction can occur by inhibiting Akt kinase in AML cells [27–29]. The results of the present study corroborate those of previous reports, suggesting the efficacy of SBL-060 against AML cells by inhibiting Akt, thus hindering the regular cell cycle and inducing cellular apoptosis.

## Conclusion

SBL-060 effectively controlled the proliferation of AML cells by inhibiting ERα and Akt kinase to induce apoptosis. The dual inhibitory efficacy of SBL-060 could be further explored to develop an effective chemotherapeutic agent against AML.

## Data Availability

The datasets generated during and/or analyzed during the current study are available from the corresponding author on reasonable request.
